# Enhanced recovery in type A aortic dissection evaluating the efficacy and feasibility of early myocardial reperfusion

**DOI:** 10.3389/fcvm.2024.1520827

**Published:** 2025-01-09

**Authors:** Tianyu Niu, Zhishuo Liu, Yu Liu, Zhonglu Yang, Yuguang Ge, Lu Wang, Lin Xia, Hui Jiang

**Affiliations:** ^1^ Department of Cardiovascular Surgery, General Hospital of Northern Theater Command, Shenyang, China; ^2^ Graduate School of Dalian Medical University, Dalian, China

**Keywords:** type A aortic dissection, early myocardial reperfusion, cardioplegic arrest, perioperative outcomes, cardiovascular surgery

## Abstract

**Background:**

This study investigates the feasibility and early outcomes of early myocardial reperfusion in patients with type A aortic dissection (TAAD), evaluating its effectiveness and potential benefits compared to traditional cardioplegic arrest techniques.

**Methods:**

A retrospective analysis was conducted on 168 patients diagnosed with TAAD who underwent surgery at the General Hospital of the Northern Theater Command in China from January 2021 to July 2024. Patients were divided into two groups: early myocardial reperfusion (EMR group, *n* = 66) and cardioplegic arrest (CA group, *n* = 102). Perioperative outcomes were compared between the groups.

**Results:**

Early myocardial reperfusion significantly reduced ventilation time 23.08 (18.21, 66.74) hours vs. 48.58 (19.18, 122.97) hours, *P* < 0.05], ICU stay time [58.80 (21.20, 126.68) hours vs. 84.86 (41.12, 168.81) hours, *P* < 0.05], and hospitalization time [13.00 (10.00, 16.00) days vs. 15.00 (11.75, 19.00) days, *P* < 0.05] compared to the CA group. There was no significant difference in hospital costs, first-hour chest tube drainage, left ventricular ejection fraction, or postoperative adverse events between the groups, except for the rate of CRRT treatment, where the EMR group had significantly fewer patients requiring postoperative CRRT (10.6% vs. 23.5%, *P* < 0.05).

**Conclusion:**

This study demonstrates that early myocardial reperfusion is a feasible and effective technique for TAAD, offering considerable advantages in reducing ventilation time, ICU stay, hospitalization duration and postoperative renal insufficiency.

## Introduction

Type A aortic dissection (TAAD) is a critical cardiovascular emergency that requires immediate surgical or endovascular intervention due to its high mortality rate (1). Surgical approaches to TAAD have significantly evolved, focusing on innovative techniques to improve patient outcomes. Traditionally, open-heart surgery involving deep hypothermic circulatory arrest has been common, but it carries substantial risks of myocardial ischemia, brain ischemic injury, and other complications (2, 3). Recently, minimally invasive techniques have emerged as promising alternatives, enhancing surgical efficacy and patient recovery (4, 5). These advancements aim to minimize postoperative complications and improve overall survival rates, underscoring the need for continuous exploration of more effective surgical strategies.

Our surgical team has made notable progress in developing minimally invasive techniques for the treatment of TAAD. By integrating Sun's Procedure with upper hemisternotomy, bilateral selective antegrade cerebral perfusion (bSACP), and lower body perfusion (LBP), we have successfully achieved favorable outcomes, including effective cerebral protection (6–9). Despite these advancements, there remains significant potential for further improving myocardial protection, particularly in reducing ischemic and reperfusion injuries. Exploring novel approaches and refining existing techniques are essential for optimizing surgical results and patient recovery. One promising approach is the early myocardial reperfusion technique, which maintains continuous heart activity during surgery, thereby minimizing myocardial ischemia (10, 11). This technique has potential advantages in improving patient outcomes. However, its application in aortic surgery remains underexplored (12, 13). Thus, this study aims to compare the effectiveness of cardioplegic arrest and early myocardial reperfusion techniques in upper hemisternotomy for TAAD. The focus is on myocardial protection and the potential to improve minimally invasive surgical strategies. This comparison will offer valuable insights into optimizing clinical practices and improving patient outcomes in TAAD treatment.

## Methods

### Patients

**Figure 1:**
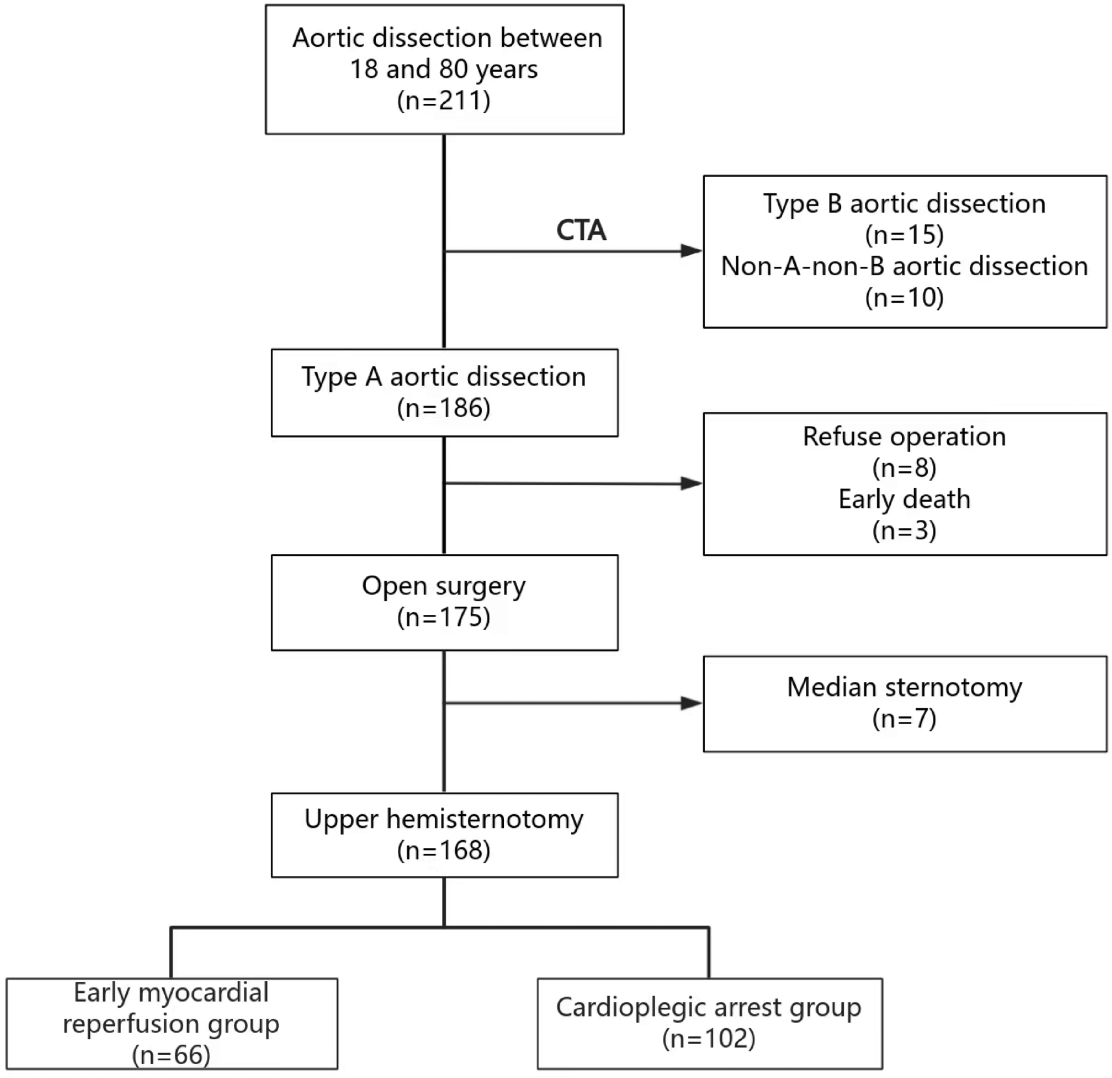
Patient flow diagram.

Approval for this study was granted by the Institutional Ethics Research Board (Protocol #K-2020019). Individual patient consent was not required due to the retrospective nature of the cohort study. The patient flow diagram is shown in Figure 1. A total of 211 consecutive patients were diagnosed with aortic dissection at the General Hospital of the Northern Theater Command from January 2021 to July 2024. Of these, 15 patients were diagnosed with type B aortic dissection, and 10 patients with non-A-non-B aortic dissection via thoracic and abdominal aortic CTA. Eight patients declined surgical treatment, and 3 patients died before surgery due to dissection rupture. Additionally, 7 patients underwent median sternotomy for other cardiovascular diseases. Ultimately, 168 patients were included in the study cohort and were divided into two groups: 66 patients in the early myocardial reperfusion group (EMR group) and 102 patients in the cardioplegic arrest group (CA group). Inclusion criteria included age between 18 and 80 years old, confirmed diagnosis of TAAD via thoracic and abdominal aortic CTA, and a preoperative cardiac ultrasound examination confirming the condition of the aortic valve and the absence of pericardial tamponade. Exclusion criteria were failure to meet the inclusion criteria, a history of two or more cardiac surgeries, unstable hemodynamic conditions, the need for median thoracotomy surgery, the presence of cancer, and incomplete clinical data.

### Surgical technique

The surgical approach and cardiopulmonary bypass (CPB) strategy utilized for all patients followed the upper hemisternotomy technique for TAAD, with corresponding enhancements implemented. All patients underwent tracheal intubation and general anesthesia, with brain protection provided by ice caps during surgery. Continuous monitoring included bilateral radial artery pressure, dorsalis pedis artery pressure, cerebral oxygen saturation, nasopharyngeal temperature, and rectal temperature.

The surgical procedure involved a sternotomy from the suprasternal fossa to the center of the fourth intercostal space, followed by a horizontal cut of the right half of the sternum at the fourth intercostal space. The innominate vein, brachiocephalic artery, left common carotid artery, and left subclavian artery were individually isolated. After systemic heparinization, the surgeon selected the appropriate arterial cannulation site based on the extent of the dissection and performed direct right atrial cannulation for venous access. CPB was established through arterial and venous cannulation, with a left heart drainage tube placed at the root of the right upper pulmonary vein.

For patients in the CA group, after establishing CPB, the nasopharyngeal temperature was gradually reduced to 32°C. The aorta was clamped, and the ascending aorta was transected. Cardiac arrest was induced by direct perfusion through the coronary sinus. Procedures such as aortic valve reconstruction, aortic valve replacement, and Bentall surgery were performed as needed based on the involvement of the AD.

The left common carotid artery and left subclavian artery were clamped using Hem-o-lock clips, and the aortic arch was excised. A frozen elephant trunk (FET) was inserted into the descending aorta, and LBP was initiated using a 16Fr cannula with an occlusion balloon (Longlaifu, Changzhou, China) inserted through the distal artery of the 4-branch prosthetic graft (Hemashield Platinum Double Velour Vascular Graft; MAQUET, La Ciotat, France), providing blood flow recovery to the lower body at a rate of 25 ml/kg·min. Once the distal aorta incorporating the stent graft was securely attached to the distal end of the 4-branch prosthetic graft, LBP was initiated via the perfusion limb of the 4-branch prosthetic graft. A four-branch prosthetic graft was anastomosed with the FET and distal aorta, and then sequentially anastomosed with the left common carotid artery, proximal aortic stump, left subclavian artery, and brachiocephalic artery. After completing the anastomosis of the left common carotid artery, CPB gradually returned to normal flow, and rewarming was initiated.

In the EMR group, after the establishment of CPB and cardiac arrest, the aortic root was addressed based on the extent of aortic dissection involvement. Once the 4-branch prosthetic graft was anastomosed to the proximal ascending aorta, a perfusion tube was inserted into the graft, the distal end was clamped, and selective myocardial perfusion was initiated. Warm blood (at a flow rate of 250–400 ml and maintained at a temperature of approximately 32°C) was used to achieve early cardiac resuscitation. The perfusion flow rate was adjusted based on electrocardiogram (ECG) monitoring of ST-segment changes and perfusion pressure, which was maintained at approximately 150 mmHg using a myocardial protection perfusion device with built-in pressure monitoring. Effective left ventricular drainage was ensured during selective myocardial perfusion, with the drainage volume closely matching the perfusion flow rate to prevent ventricular distension. Following this, the aortic arch was excised, and the FET was inserted. The remaining steps of the procedure were identical to those performed in the CA group.

### Statistical analysis

This study utilized SPSS Statistics 27.0 (IBM Corporation, Chicago, USA) and R version 4.4.1 (R Foundation for Statistical Computing, Vienna, Austria) for data analysis. Categorical variables were presented as counts and percentages (%), and group comparisons were conducted using either the chi-square test or Fisher's exact test. Continuous variables following a normal distribution were expressed as mean ± standard deviation, while those not following a normal distribution were presented as median and interquartile range, with group comparisons performed using *t*-tests or Mann-Whitney *U*-tests. We utilized the standardized mean difference (SMD) to evaluate the balance between groups. An SMD value of less than 0.1 indicates that the baseline characteristics between the two groups are well balanced. A generalized linear model was used to analyze the repeated measurement data, as they did not follow a normal distribution in this study. A *P*-value of less than 0.05 was considered statistically significant. GraphPad Prism 10 (GraphPad Software Inc., San Diego, CA, USA) was used for generating graphical illustrations.

## Results

### Preoperative characteristics

No significant differences were observed between the two groups regarding age, sex, weight, time of onset, hypertension, smoke, drink and Marfan's disease. The comparisons were as follows: age [49.27 ± 12.33 years (EMR group) vs. 52.04 ± 11.58 years (CA group)], sex [78.8% male (EMR group) vs. 67.6% male (CA group)], weight [80.00 kg (70.00, 95.00) (EMR group) vs. 79.00 kg (67.50, 90.00) (CA group)], time of onset [70.00 h (28.75, 160.75) (EMR group) vs. 48.50 h (22.75, 106.50) (CA group)], smoke [45.5% (EMR group) vs. 39.2% (CA group)], drink [27.3% (EMR group) vs. 23.5% (CA group)], hypertension prevalence [62.1% (EMR group) vs. 70.6% (CA group)], coronary heart disease prevalence [7.6% (EMR group) vs. 9.8% (CA group)] and Marfan's disease prevalence [7.6% (EMR group) vs. 5.9% (CA group)]. In the EMR group, no patients had diabetes and a history of cerebrovascular accidents (CVA), while in the CA group, 4 patients (3.9%) had a history of CVA and 5 patients (4.9%) had a history of CVA. There were 5 patients (7.6%) EMR group had cerebral malperfusion, 1 patient (1.5%) had renal malperfusion and 2 patients (3.0%) had limb malperfusion in EMR group at the time of admission. And 5 patients (4.9%) had cerebral malperfusion, 1 patient (1.0%) had visceral malperfusion, 7 patient (6.9%) had renal malperfusion and 7 patients (6.9%) had limb malperfusion at the time of admission in CA group. The preoperative characteristics of patients are detailed in Table 1.

**Table 1:** Patient demographics and preoperative data.

	EMR group	CA group	*P*-value
No. of Patients (*n*, %)	66 (39.2%)	102 (60.7%)	
Age (years, *x¯* ± *s*)	49.27 ± 12.33	52.04 ± 11.58	0.142
Male (*n*, %)	52 (78.8%)	69 (67.6%)	0.116
Weight (kg)	80.00 (70.00, 95.00)	79.00 (67.50, 90.00)	0.273
Time of onset (h)	70.00 (28.75, 160.75)	48.50 (22.75, 106.50)	0.147
Smoker (*n*, %)	30 (45.5%)	40 (39.2%)	0.423
Drunkr (*n*, %)	18 (27.3%)	24 (23.5%)	0.584
Hypertension (*n*, %)	41 (62.1%)	72 (70.6%)	0.253
Diabetes (*n*, %)	0 (0.0%)	4 (3.9%)	0.155
History of CVA (*n*, %)	0 (0.0%)	5 (4.9%)	0.158
Coronary heart disease (*n*, %)	5 (7.6%)	10 (9.8%)	0.621
Marfan's disease (*n*, %)	5 (7.6%)	6 (5.9%)	0.665
Bicuspid aortic valve (*n*, %)	3 (4.5%)	7 (6.9%)	0.535
Moderate and severe aortic regurgitation (*n*, %)	25 (37.9%)	44 (43.1%)	0.499
Malperfusion (*n*, %)			
Cerebral malperfusion	5 (7.6%)	5 (4.9%)	0.474
Visceral malperfusion	0 (0.0%)	1 (1.0%)	1.000
Renal malperfusion	1 (1.5%)	7 (6.9%)	0.149
Limb malperfusion	2 (3.0%)	7 (6.9%)	0.281

EMR, early myocardial reperfusion; CA, cardioplegic arrest; CVA, cerebrovascular accident.

### Intraoperative data

Table 2 presents the surgical procedures and intraoperative data of the patients, showing no significant differences between the two groups except for the time of cardioplegic arrest [45.00 (36.00, 73.00) (EMR group) minutes vs. 85.00 (71.00, 101.25) (CA group) minutes]. The CPB time was comparable between the EMR group and the CA group [157.00 (135.75, 190.50) minutes vs. 162.00 (137.00, 198.25) minutes], as were the cross-clamp time [92.00 (78.00, 112.75) vs. 92.00 (80.00, 106.00) minutes] and circulatory arrest time [6.00 (5.00, 7.00) minutes vs. 6.00 (5.00, 7.00) minutes]. Additionally, both groups primarily utilized bilateral selective antegrade cerebral perfusion as their brain perfusion strategy (95.5% vs. 97.1%).

**Table 2:** Surgical procedure and intraoperative data.

	EMR group	CA group	*P*-value
Aortic root repair (*n*, %)			
Aortic valvuloplasty	43 (65.2%)	61 (59.8%)	0.486
Aortic valve replacement	1 (1.5%)	4 (3.9%)	0.649
Bentall procedure	3 (4.5%)	12 (11.8%)	0.109
David procedure	2 (3.0%)	8 (7.8%)	0.198
Ascending aorta replacement (*n*, %)	17 (25.8%)	17 (16.7%)	0.152
Total arch replacement & FET (*n*, %)	66 (100.0%)	99 (97.1%)	0.280
Concomitant procedures (*n*, %)			
Vertebral artery reconstruction	4 (6.1%)	3 (2.9%)	0.435
Arterial perfusion position (*n*, %)			
Innominate artery	49 (74.2%)	78 (75.5%)	0.743
Left common carotid artery	10 (15.2%)	13 (12.7%)	0.658
Right common carotid artery	3 (4.5%)	1 (1.0%)	0.301
Right subclavian artery	2 (3.0%)	2 (2.0%)	1.000
Femoral artery	1 (1.5%)	5 (4.9%)	0.405
CPB time (min)	157.00 (135.75, 190.50)	162.00 (137.00, 198.25)	0.385
Crossclamp time (min)	92.00 (78.00, 112.75)	92.00 (80.00, 106.00)	0.797
Cardioplegic arrest time (min)	45.00 (36.00, 73.00)	85.00 (71.00, 101.25)	<0.001
Circulatory arrest time (min)	6.00 (5.00, 7.00)	6.00 (5.00, 7.00)	0.214
SCP (*n*, %)			
Selective antegrade cerebral perfusion	66 (100.0%)	101 (99.0%)	1.000
Bilateral selective antegrade cerebral perfusion	63 (95.5%)	99 (97.1%)	0.681
Spontaneous heart rebeating (*n*, %)	55 (83.3%)	83 (81.4%)	0.746

EMR, early myocardial reperfusion; CA, cardioplegic arrest; FET, frozen elephant trunk; CPB, cardiopulmonary bypass; SCP, selective cerebral perfusion.

### Early postoperative outcomes

The early postoperative outcomes between the EMR group and CA group are presented in Table 3. The ventilation time [23.08 (18.21, 66.74) hours vs. 48.58 (19.18, 122.97) hours], ICU stay time [58.80 (21.20, 126.68) hours vs. 84.86 (41.12, 168.81) hours], and hospitalization time [13.00 (10.00, 16.00) days vs. 15.00 (11.75, 19.00) days] were significantly shorter in the EMR group compared to the CA group (*P* < 0.05). There was no significant difference in hospital costs [22.59 (20.67, 25.12) ¥ vs. 23.16 (21.18, 28.18) ¥], first-day chest tube drainage [245.00 (150.00, 352.50) ml vs. 250.00 (167.50, 332.50) ml], left ventricular ejection fraction [58.00 (56.50, 59.00) % vs. 58.00 (56.00, 59.00) %] and blood transfusion (83.3% vs. 89.2%). The units of red blood cell transfusion were significantly lower in the EMR group compared with the CA group [2.00 (0.00, 3.85) ml vs. 3.25 (0.75, 6.00) U]. The postoperative adverse events between the two groups had no significant difference, except for the rate of CRRT treatment, where the EMR group had significantly fewer patients requiring postoperative CRRT (10.6% vs. 23.5%).

**Table 3:** Early postoperative outcomes.

	EMR group	CA group	*P*-value
Ventilation time (h)	23.08 (18.21, 66.74)	48.58 (19.18, 122.97)	0.035
ICU stay time (h)	58.80 (21.20, 126.68)	84.86 (41.12, 168.81)	0.013
Hospitalization time (d)	13.00 (10.00, 16.00)	15.00 (11.75, 19.00)	0.020
hospital costs (¥10,000)	22.59 (20.67, 25.12)	23.16 (21.18, 28.18)	0.095
The first day chest tube drainage (ml)	245.00 (150.00, 352.50)	250.00 (167.50, 332.50)	0.884
left ventricular ejection fraction (%)	58.00 (56.50, 59.00)	58.00 (56.00, 59.00)	0.835
blood transfusion (*n*, %)	55 (83.3%)	91 (89.2%)	0.270
Red blood cell transfusion (U)	2.00 (0.00, 3.85)	3.25 (0.75, 6.00)	0.005
ECMO (*n*, %)	0 (0.0%)	1 (1.0%)	1.000
CRRT (*n*, %)	7 (10.6%)	24 (23.5%)	0.035
Reoperation for hemostasis (*n*, %)	1 (1.5%)	1 (1.0%)	1.000
Reventilation (*n*, %)	3 (4.5%)	6 (5.9%)	0.707
Tracheostomy (*n*, %)	1 (1.5%)	3 (2.9%)	0.655
Spinal cord injury (*n*, %)	3 (4.5%)	5 (4.9%)	1.000
Lumbar puncture (*n*, %)	4 (6.0%)	6 (5.9%)	0.962
Stroke (*n*, %)	1 (1.5%)	5 (4.9%)	0.405
TND (*n*, %)	2 (3.0%)	5 (4.9%)	0.705
Poor wound healing (*n*, %)	1 (1.5%)	3 (2.9%)	0.655
Hospital mortality (*n*, %)	2 (3.0%)	8 (7.8%)	0.198

EMR, early myocardial reperfusion; CA, cardioplegic arrest; ICU, intensive care unit; ECMO, extracorporeal membrane oxygenation; CRRT, continuous renal replacement therapy; TND, transient neurological dysfunction.

Ten patients died during hospitalization. In the EMR group, two patients died of multiple organ failure. In the CA group, two patients died of multiple organ failure, three patients died of circulatory dysfunction, and three patients died of malignant arrhythmia.

### Perioperative laboratory data

**Figure 2:**
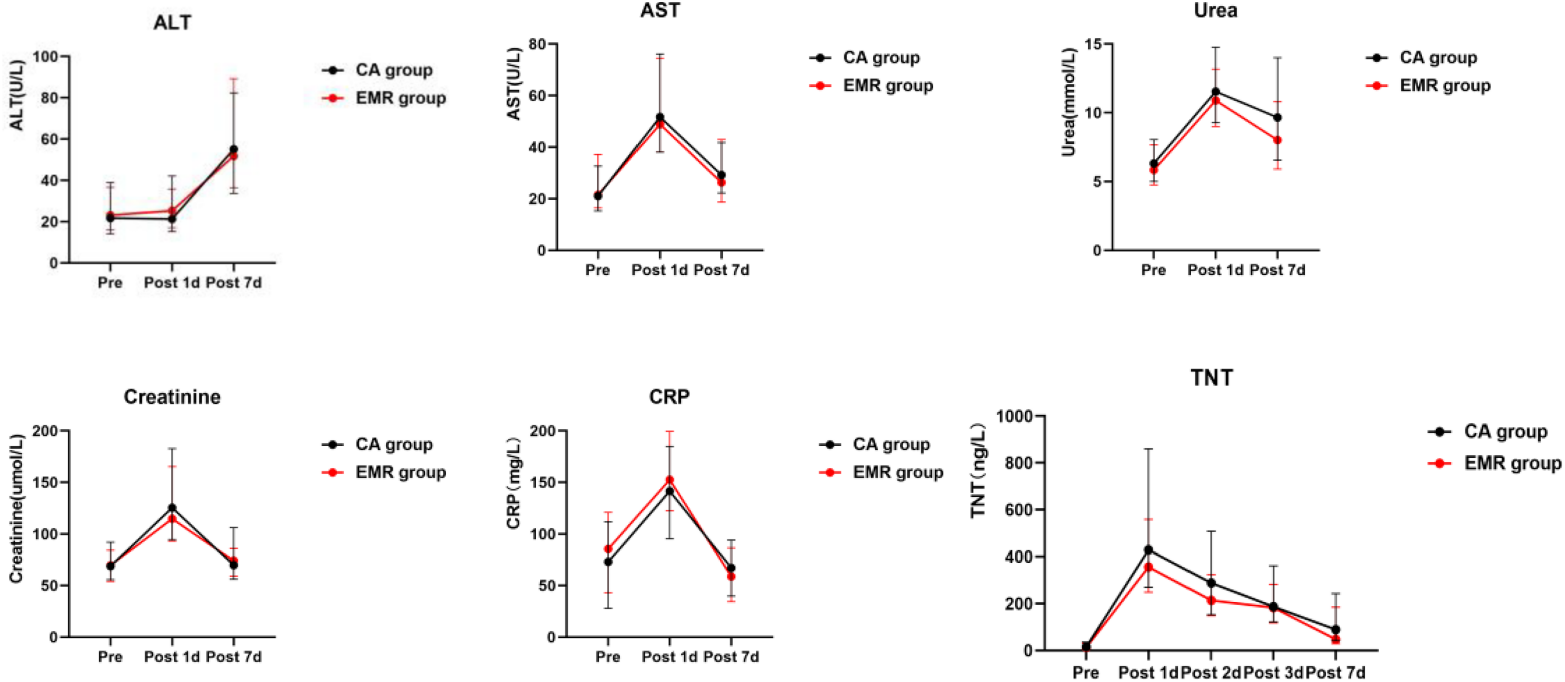
The trend of laboratory outcomes in patients before and after surgery. EMR, early myocardial reperfusion; CA, cardioplegic arrest; ALT, alanine transaminase; AST, aspartate aminotransferase; TNT, troponin T; CRP, C-reactive protein.

The trend of laboratory outcomes in patients before and after surgery is shown in Tables 4, 5 and Figure 2. There was a significant difference in TNT levels between the two groups of patients 1 week after surgery [47.00 (29.00, 184.75) ng/L vs. 89.00 (41.50, 243.00) ng/L, *P* = 0.009]. However, there were no significant differences in other laboratory results (*P* > 0.05). In addition, there was no significant difference in the group comparison of all the above laboratory outcomes, but significant differences were observed in the intra-group comparison (*P* < 0.05).

**Table 4:** The laboratory outcomes of two groups at different time points for treatment.

	EMR group	CA group	*P*-value
ALT (U/L)			
Preoperative	23.11 (15.65, 36.71)	21.28 (13.43, 42.16)	0.409
1 day after surgery	25.31 (17.07, 35.82)	21.25 (15.20, 42.16)	0.350
7 day after surgery	51.78 (36.45, 89.20)	55.14 (33.73, 82.36)	0.647
AST (U/L)			
Preoperative	21.52 (16.40, 38.46)	21.03 (15.22, 32.63)	0.452
1 day after surgery	48.88 (38.09, 74.56)	51.68 (38.01, 76.19)	0.642
7 day after surgery	26.31 (18.71, 42.96)	29.22 (22.20, 41.76)	0.112
Urea (mmol/L)			
Preoperative	5.84 (4.73, 7.58)	6.32 (5.08, 7.91)	0.223
1 day after surgery	10.89 (9.00, 13.16)	11.54 (9.28, 14.77)	0.175
7 day after surgery	8.01 (5.91, 10.79)	9.65 (6.54, 14.00)	0.081
Creatinine (mmol/L)			
Preoperative	69.60 (55.44, 84.47)	69.57 (56.02, 93.67)	0.892
1 day after surgery	114.71 (93.27, 165.20)	126.84 (94.69, 183.96)	0.289
7 day after surgery	74.13 (59.05, 86.30)	69.80 (56.02, 106.28)	0.814
TnT (ng/L)			
Preoperative	13.00 (6.00, 31.00)	15.50 (8.00, 39.00)	0.127
1 day after surgery	361.00 (248.75, 572.50)	429.00 (269.50, 859.50)	0.177
2 day after surgery	214.00 (149.00, 323.50)	282.00 (153.50, 500.50)	0.065
3 day after surgery	182.50 (118.00, 281.75)	187.00 (122.00, 361.00)	0.428
7 day after surgery	47.00 (29.00, 184.75)	89.00 (41.50, 243.00)	0.009
CRP (mg/L)			
Preoperative	85.55 (43.16, 120.95)	76.09 (24.46, 112.52)	0.128
1 day after surgery	152.55 (122.65, 199.43)	141.60 (95.39, 184.91)	0.124
7 day after surgery	58.76 (34.43, 86.65)	67.07 (39.69, 94.12)	0.440

EMR, early myocardial reperfusion; CA, cardioplegic arrest; ALT, alanine transaminase; AST, aspartate aminotransferase; TNT, troponin T; CRP, C-reactive protein.

**Table 5:** Group comparison of laboratory outcomes of two groups at different time points for treatment.

Biomakers	Group	*N*	*x¯* ± *s*	Wald *X*^2^	*P*-value
ALT (U/L)					
Group comparison	CA group	102	56.73 ± 7.73	0.311	0.577
	EMR group	66	51.35 ± 5.76		
Intragroup comparation	Preoperative	168	35.65 ± 3.83	37.896	<0.001
	1 day after surgery	167	53.55 ± 10.91		
	7 day after surgery	167	72.91 ± 4.83		
AST (U/L)					
Group comparison	CA group	102	78.31 ± 20.26	1.403	0.236
	EMR group	66	51.85 ± 9.27		
Intragroup comparation	Preoperative	168	38.47 ± 6.86	6.776	0.034
	1 day after surgery	167	122.74 ± 32.87		
	7 day after surgery	166	34.02 ± 3.29		
Urea (mmol/L)					
Group comparison	CA group	102	10.27 ± 0.38	2.975	0.085
	EMR group	66	9.24 ± 0.45		
Intragroup comparation	Preoperative	168	7.07 ± 0.30	334.305	<0.001
	1 day after surgery	167	11.90 ± 0.31		
	7 day after surgery	167	10.29 ± 0.44		
Creatinine (mmol/L)					
Group comparison	CA group	102	111.54 ± 6.13	0.471	0.492
	EMR group	66	104.17 ± 8.82		
Intragroup comparation	Preoperative	168	85.52 ± 5.82	197.963	<0.001
	1 day after surgery	167	146.58 ± 6.17		
	7 day after surgery	167	91.47 ± 6.08		
TNT (ng/L)					
Group comparison	CA group	102	546.64 ± 124.53	2.185	0.139
	EMR group	66	319.67 ± 89.85		
Intragroup comparation	Preoperative	168	23.55 ± 18.86	155.299	<0.001
	1 day after surgery	167	774.68 ± 101.66		
	2 day after surgery	167	558.63 ± 102.59		
	3 day after surgery	167	453.28 ± 102.93		
	7 day after surgery	167	355.63 ± 94.19		
CRP (mg/L)					
Group comparison	CA group	102	97.74 ± 3.91	1.682	0.195
	EMR group	66	105.98 ± 4.99		
Intragroup comparation	Preoperative	168	84.62 ± 4.83	361.893	<0.001
	1 day after surgery	167	152.38 ± 4.72		
	7 day after surgery	167	68.58 ± 3.06		

EMR, early myocardial reperfusion; CA, cardioplegic arrest; ALT, alanine transaminase; AST, aspartate aminotransferase; TNT, troponin T; CRP, C-reactive protein.

## Discussion

This study retrospectively presents the early outcomes of early myocardial reperfusion via single upper hemisternotomy in 168 patients with TAAD from January 2021 to July 2024. The results indicate that early myocardial reperfusion can significantly reduce ventilation time, ICU stay time, hospitalization time, the quantity of red blood cell transfusion and the TNT levels of patients 1 week after surgery. In this study, selective myocardial perfusion was performed to enable early heart activity following aortic root repair. The findings suggest that this technique may shorten myocardial ischemia duration, reduce myocardial reperfusion injury, and ultimately help protect heart tissue from ischemic damage.

Our study found that EMR technology can reduce the postoperative CRRT in patients. This may be due to the role of early myocardial reperfusion in reducing ischemic injury (14). Improved cardiac output and better hemodynamic stability may have contributed to a decreased risk of renal hypoperfusion and subsequent acute kidney injury, reducing the need for CRRT.

In this study, three patients in the CA group died from circulatory dysfunction (3%) and three died from malignant arrhythmias (3%), while there were no acardiac deaths in the EMR group. This may be due to the fact that EMR technology reduces myocardial damage and protects heart function to reduce the occurrence of cardiac death. Although there was no significant difference in mortality between the two groups in our study, beating hearts have been shown in other studies to have better 6-year survival and lower cardiogenic mortality than cardiac arrest surgery (10).

In this study, with no difference in baseline characteristics between the two groups, the EMR group resumed myocardial perfusion after the completion of proximal aortic treatment, significantly reducing the duration of intraoperative cardiac arrest. The main purpose of this technology is to protect the myocardium, reduce the damage caused by ischemia and hypoxia to the heart, protect the heart function, so as to protect the function of the patients' important organs and reduce postoperative complications.

However, since this surgical strategy is a newly developed surgical technique in our centerand has only been recently implemented, the sample size is small and the medium and long-term follow-up results are lacking. In the future, we will consider conducting multi-center randomized controlled studies with larger sample sizes in conjunction with other centers to evaluate the feasibility and safety of this technology.

Multivariate regression analysis could provide additional insights by adjusting for residual confounders and further validating the observed outcomes. While the current sample size limits the statistical power for such an analysis, this as a limitation of our study. We will incorporate multivariate regression analysis as an essential component of future, larger-scale studies to strengthen the reliability of our findings.

Research has shown that beating heart surgery offers several advantages over traditional methods. For instance, it is associated with less intraoperative bleeding, reduced release of myocardial injury markers, shortened ventilation and ICU stay times, and a decreased risk of complications (15, 16). Ma et al. demonstrated that during beating heart surgery, myocardial oxygen consumption decreased, blood supply increased, and coronary blood circulation remained close to physiological levels, thereby reducing the postoperative release of cTnI and myocardial reperfusion injury (17). However, they noted that beating heart technique might increase the risk of air embolism. Servet et al. found that using the beating heart technique during infant aortic arch reconstruction resulted in a lower incidence of postoperative acute renal failure and delayed sternal closure (18). Andreas et al. suggested that early myocardial reperfusion significantly reduces the duration of CPB and myocardial ischemia time and lowers the incidence of cardiac output syndrome, although it is associated with increased intraoperative bleeding (19). These variations may be due to individual differences among patients, surgical strategies, and surgeon experience. Wang et al. reported favorable outcomes with thoracoscopic closure of atrial septal defects on perfused beating hearts, highlighting its efficacy and safety in avoiding ischemic injury (20). Similarly, comparative studies in pediatric extracardiac total cavopulmonary connection have shown reduced complication rates and improved recovery with beating heart techniques compared to cardioplegic arrest (21). In adult cardiac surgery, Chen et al. demonstrated that for isolated tricuspid valve operations, beating heart techniques contributed to improved outcomes, emphasizing the potential advantages of avoiding prolonged ischemia (22).

However, heart beating surgery may increase patients' risk of air embolism, this condition that greatly increases risk of intraoperative death (23). In our surgery, we used meticulous de-airing techniques, antegrade perfusion to facilitate air clearance, and continuous intraoperative transesophageal echocardiography monitoring to detect and resolve residual air in real-time. We have also emphasized the importance of a multidisciplinary approach involving perfusionists, anesthesiologists, and surgeons to ensure optimal patient safety during EMR procedures.

In our study, the *P*-value for hospital cost differences between the EMR and CA groups was 0.095, indicating marginal statistical significance. We believe that this result may be attributed to the relatively small sample size, which limits the statistical power to detect significant differences. Additionally, potential selection bias inherent in the retrospective study design could have influenced this finding. Another possible explanation is that while the reduced ICU and hospital stay times in the EMR group may decrease some costs, other factors—such as the initial costs associated with implementing a novel surgical technique like EMR—might offset these savings. For example, equipment requirements for early myocardial reperfusion could contribute to higher upfront costs, balancing out the reduced expenditure from shorter hospital stays.

In summary, this study provides a strong evidence for the efficacy of the early myocardial reperfusion technique in TAAD surgery. This method can theoretically reduce myocardial ischemia time, myocardial reperfusion injury, and the occurrence of postoperative cardiac adverse events. By maintaining continuous heart activity during surgery, the early myocardial reperfusion technique offers substantial benefits in terms of myocardial protection and overall patient outcomes. This approach represents a promising direction for improving surgical outcomes in TAAD patients and warrants further exploration and refinement in clinical practice.

### Limitations

This study has several limitations. First, it is a retrospective, single-center analysis with a small sample size, introducing a risk of bias. Second, due to individual differences among patients and preoperative comorbidities, achieving randomization was challenging, potentially leading to selection and observational biases. However, due to our current dataset's constraints, this study lacks adjustment for relevant baseline and operative covariates and our study does not incorporate multivariate analysis to adjust for potential confounding variable. Third, the study only compared perioperative surgical outcomes and lacked mid-term and long-term follow-up data. Finally, our study only used TNT as an indicator to measure the severity of myocardial injury and lacked other biomarkers. Additionally, the young age of patients included in the cohort and their very low prevalence of malperfusion suggest that there could be confounders introducing bias in this analysis. However, we believe that these results are particularly relevant and applicable to younger patient populations. In the future, we plan to conduct a prospective randomized controlled trial with a larger sample size, multicenter, long-term follow-up and to examine multiple markers of myocardial injury postoperatively to evaluate the safety and feasibility of this surgical technique over the long term.

## Conclusion

This study demonstrates that early myocardial reperfusion is feasible for TAAD and yields significant surgical benefits. However, this technique demands advanced technical skills, extensive anatomical knowledge, and substantial surgical experience from the surgeon. Overall, early myocardial reperfusion surgery represents a significant technological advancement in the field of cardiovascular surgery, offering a safer and more effective treatment option for patients with major artery dissection.

## Data Availability

The raw data supporting the conclusions of this article will be made available by the authors, without undue reservation.
