# Supplementary material for: Enhanced recovery in type A aortic dissection evaluating the efficacy and feasibility of early myocardial reperfusion
Source: Front Cardiovasc Med. 2025 Jan 9;11:1520827. doi: 10.3389/fcvm.2024.1520827 (PMC11754212; doi:10.3389/fcvm.2024.1520827)
Supplement: Supplementary file 1 [file Table1.pdf]

Supplementary Table 1 SMD of baseline feature

		SMD
Age (years, $\bar{x} \pm s$)		0.031
Male (n, %)		0.054
Weight(kg)		0.088
Time of onset (hours)		0.026
Smoker (n, %)		0.027
Drunkr (n, %)		0.086
Hypertension(n, %)		0.080
Diabetes (n, %)		0.086
History of CVA (n, %)		0.021
Coronary heart disease (n, %)		0.079
Marfan's disease (n, %)		0.068
Bicuspid aortic valve (n, %)		0.010
Moderate and severe aortic regurgitation (n, %)		0.010
Malperfusion (n, %)	Cerebral malperfusion	0.011
	Visceral malperfusion	0.041
	Renal malperfusion	0.030
	Limb malperfusion	0.077
Aortic root repair (n, %)	Aortic valvuloplasty	0.011
	Aortic valve replacement	0.048
	Bentall procedure	0.066
	David procedure	0.013
Ascending aorta replacement (n, %)		0.024
Total arch replacement & FET (n, %)		0.046
Concomitant procedures (n, %)	Vertebral artery reconstruction	0.015
Arterial perfusion position (n, %)	Innominate artery	0.029
	Left common carotid artery	0.069
	Right common carotid artery	0.021
	Right subclavian artery	0.070

	Femoral artery	0.093

Abbreviations: *CVA*, cerebrovascular accident; *FET*, frozen elephant trunk; *SMD*, standardized mean difference
